# Bacteriophage therapy against *Pseudomonas aeruginosa* biofilms: a review

**DOI:** 10.1186/s12941-020-00389-5

**Published:** 2020-09-30

**Authors:** Zahra Chegini, Amin Khoshbayan, Majid Taati Moghadam, Iman Farahani, Parham Jazireian, Aref Shariati

**Affiliations:** 1 Department of Microbiology, School of Medicine, Iran University of Medical Sciences, Tehran, Iran; 2 Student Research Committee, Iran University of Medical Sciences, Tehran, Iran; 3 Molecular and Medicine Research Center, Department of Microbiology and Immunology, School of Medicine, Arak University of Medical Sciences, Arak, Iran; 4 Department of Genetics, Reproductive Biomedicine Research Center, Royan Institute for Reproductive Biomedicine, ACECR, Tehran, Iran; 5 Department of Microbiology, School of Medicine, Shahid Beheshti University of Medical Sciences, Tehran, Iran

**Keywords:** MDR *P. aeruginosa*, Biofilm, Bacteriophage, Antibiofilm effects

## Abstract

Multi-Drug Resistant (MDR) *Pseudomonas aeruginosa* is one of the most important bacterial pathogens that causes infection with a high mortality rate due to resistance to different antibiotics. This bacterium prompts extensive tissue damage with varying factors of virulence, and its biofilm production causes chronic and antibiotic-resistant infections. Therefore, due to the non-applicability of antibiotics for the destruction of *P. aeruginosa* biofilm, alternative approaches have been considered by researchers, and phage therapy is one of these new therapeutic solutions. Bacteriophages can be used to eradicate *P. aeruginosa* biofilm by destroying the extracellular matrix, increasing the permeability of antibiotics into the inner layer of biofilm, and inhibiting its formation by stopping the quorum-sensing activity. Furthermore, the combined use of bacteriophages and other compounds with anti-biofilm properties such as nanoparticles, enzymes, and natural products can be of more interest because they invade the biofilm by various mechanisms and can be more effective than the one used alone. On the other hand, the use of bacteriophages for biofilm destruction has some limitations such as limited host range, high-density biofilm, sub-populate phage resistance in biofilm, and inhibition of phage infection via quorum sensing in biofilm. Therefore, in this review, we specifically discuss the use of phage therapy for inhibition of *P. aeruginosa* biofilm in clinical and in vitro studies to identify different aspects of this treatment for broader use.

## Introduction

*Pseudomonas aeruginosa* is a Gram-negative bacillus and one of the main opportunistic pathogens that have a leading role in nosocomial, acute, and chronic infections [1]. Infection with this pathogen leads to diseases with a high mortality rate in patients diagnosed with cystic fibrosis, cancer, severe burns, and immunocompromised patients [2, 3]. This bacterium can survive on water, different surfaces, and medical devices by using its influential binding factors such as flagella, pili, and biofilms. Thus, *P. aeruginosa* is abundant in natural and artificial environments, lakes, hospitals, and household sink drains [4].

Due to the widespread role of this bacterium in causing various infections and increasing antibiotic resistance, recently, the treatment failure has become a major global problem. *P. aeruginosa* has shown high intrinsic resistance to a range of antibiotics, including beta-lactams, fluoroquinolones, and aminoglycosides, which results in significant morbidity and mortality rates [5, 6]. According to U.S. Centers for Disease Control and Prevention report, it is estimated that approximately 51,000 healthcare-associated infections caused by *P. aeruginosa* occur in the United States each year, and 13% of these infections are multidrug-resistant (MDR), with roughly 400 deaths per year attributed to such infections [7, 8]. The main mechanisms of these resistances are low antibiotic permeability of the outer membrane, chromosomally encoded AmpC, and drug efflux via multi-drug efflux (Mex) systems [9]. In addition to intrinsic resistance, *P. aeruginosa* has different mechanisms for resistance to various antibiotics, such as horizontal gene transfer and mutation-driven resistance [6, 10, 11]. Mobile genetic elements such as transposons, resistance islands, prophages, integrons, and plasmids can accommodate antibiotic resistance genes and transmit them to *P. aeruginosa,* causing MDR bacteria. For example, aminoglycoside-modifying enzymes are transported to *P. aeruginosa* via mobile genetic elements and reduce the binding affinity of the antibiotic to its target site, which is the 30_S_ ribosomal subunit. Therefore, it causes resistance to aminoglycosides [12]. Furthermore, the random mutation frequency differs between antibiotics with resistance frequencies ranging from 10^6^ to 10^9^ for individual antibiotics. The rate of mutation can increase in some situations, such as the presence of DNA-damaging agents or within growth in a biofilm [6].

*Pseudomonas aeruginosa* can bind to various surfaces and form biofilms leading to chronic infections by increasing resistance to antibiotics, disinfectants, various irradiation treatments, environmental conditions, and the immune system [3, 13, 14]. Bacterial biofilm was introduced, for the first time, in 1987 as a community of microorganisms capable of binding to surfaces and forming an exopolysaccharide and extracellular matrix [15]. Biofilms are approximately 10 to 1000 times more resistant to antibiotics than planktonic cells due to the lack of antibiotic penetration into the complex polysaccharide matrix (glycocalyx) of biofilms [16, 17]. Thus, biofilms and the inherent and acquired antibiotic resistance mechanism of *P. aeruginosa* have increased the prevalence of MDR strains in recent years with virtually no fully effective antibiotics available to stop this bacterium.

So, researchers are looking for new ways to inhibit *P. aeruginosa* biofilms. Phage therapy is one of the important methods to inhibit *P.* aeruginosa biofilm [18]. Bacteriophages are viruses that invade bacteria; they were discovered almost a century ago and are divided into two lytic (virulent phages) and temperate categories depending on their life cycle [19, 20]. After attaching to their host, the lytic phages inject their genetic materials into the host chromosome and replicate along with the host cell DNA, then disperse via the host lysis to repeat the infection cycle for other hosts. Of note, obligately lytic bacteriophages are often a matter of interest for therapeutic purposes because they lead to the killing of their bacterial host cell rapidly [21]. On the other hand, temperate phages generally integrate their genome into the host chromosome or sometimes keep it as a plasmid, which is transmitted to the daughter cells by cell division [21, 22]. Using antibiotics has always been a good solution for the treatment of bacterial infections due to their inexpensive cost and extreme effectiveness on various bacterial agents. After World War II, the widespread effective use of antibiotics diminished the interest of different societies in using bacteriophages [23, 24]. Nevertheless, over the years, for various reasons such as overuse and misuse of broad-spectrum antibiotics, bacterial resistance to the existing antibiotics increased, and MDR strains dramatically expanded worldwide. This situation forced scientists to think about reusing bacteriophages instead of antibiotics to treat bacterial infections [25, 26].

Bacteriophages that specifically target *Pseudomonas* genus were first discovered in the middle of the twentieth century, and due to the great role of this microorganism in nosocomial infections and high antibiotic resistance, using bacteriophages to inhibit *P. aeruginosa* has been highly regarded [27, 28]. The use of two or more bacteriophage mixtures with different host ranges in a single suspension as a bacteriophage cocktail is usually more effective for inhibiting bacterial infections [29, 30]. Bacteriophage cocktail causes better reduction of bacterial density and improve bacteriophages’ efficiency, and also in vitro studies have shown that bacteriophage cocktail result in a higher reduction in *P. aeruginosa* infections [31]. Bacteriophage cocktails can easily penetrate the *P. aeruginosa* biofilm and destroy its structure by inducing the synthesis of enzymes such as polysaccharide depolymerase [32]. Polysaccharide depolymerase, a polysaccharide hydrolase encoded by bacteriophages, can specifically degrade the macromolecule carbohydrates of the host bacterial envelope. This enzyme helps the bacteriophage to adsorb, invade, and disintegrate the host bacteria [33]. Furthermore, bacteriophages generate peptidoglycan hydrolases enzymes, called Endolysins, at the end of the lytic cycle. They decompose peptidoglycan from the inside and assist in forming new progeny phages to release from the cell [34]. Endolysins are always proposed as antibacterial agents because of their high specific activity and unique mode of action against bacteria. The activity of Endolysins is independent of antibiotic susceptibility patterns [35, 36]. It should be noted that bacteriophages have advantages over antibiotics to inhibit infections caused by bacterial biofilms. For example, bacteriophages penetrate the inner layer of the biofilm, unlike antibiotics that affect bacteria at the surface. Furthermore, bacteriophages are capable of infecting persister cells and destroying them if they are reactivated. They can also dissolve the biofilm matrix by producing an enzyme or induce enzyme production by the bacterial host [37–39]. Another mechanism of biofilm inhibition by bacteriophages is the production of enzymes that inhibit biofilm production. One study reported that bacteriophages can inducing synthesis of quorum quenching (QQ) lactonase by genetic modification, which inhibits biofilm formation in *P. aeruginosa* by hydrolysis of Acyl homoserine lactones (AHL) and inhibition of quorum-sensing (QS) activity [40].

Therefore, regarding the determinant role of *P. aeruginosa* biofilm in the development of antibiotic resistance and chronic infections, finding new strategies as a treatment for its inhibition is essential. In this review, we will specifically discuss the role of bacteriophages in the inhibition and destruction of *P. aeruginosa* biofilm to identify various aspects of phage therapy in this field and facilitate its possible widespread use in clinical practice.

## Phage therapy for inhibition of MDR *P. aeruginosa* biofilm: in vitro studies

Many studies have indicated that bacteriophages are one of the most promising weapons for the elimination of in vitro *P. aeruginosa* biofilms; for example, Adnan et al. used bacteriophage M-1 that was isolated from wastewater to remove biofilms caused by MDR isolates of *P. aeruginosa.* The results showed that the bacteriophage MA-1 reduced the growth rate of *P. aeruginosa* and decreased biofilms after 6 h of treatment. An important point discussed in this study was that bacteriophage can degrade alginate polymers through enzymatic activities, and even it can destroy the 20-day biofilm formed by *P. aeruginosa*. Phage can also destroy biofilms indirectly by killing bacteria before attaching, or after colonizing the surface [41]. Another study examined the effect of PB1-like, phiKZ-like, and LUZ24-like phages against MDR *P. aeruginosa* under variable growth conditions; the results indicated that each phage alone was able to suppress planktonic and biofilm form of MDR isolates. The phiKZ-like viruses were the most potent phages in the suppression of planktonic form. Besides, LUZ24-like phage was the most effective phage to destroy the biofilm of antibiotic-resistant isolates. Also, the effect of the cocktail consisting of all three phages was more potent than that of each phage alone. Researchers attributed the small size of the LUZ24-like phage to its significant effect on biofilm degradation, while a high volume phiKZ-like phage had the least destructive effect on the biofilm. Also, it has been suggested that phages may not have an excellent effect on high-density biofilms. However, they can prevent further accumulation and diffusion of biofilms by reducing migratory bacteria [42].

Fong et al. used bacteriophages Pa193, Pa204, Pa222, and Pa223 to eliminate the biofilm of *P. aeruginosa* isolated from patients with chronic rhinosinusitis and found that a single dose of these phages alone and in cocktail significantly decreased the rate of biofilm production after 24 and 48 h of treatment. Although single phages reduced 53–73% of the biofilms of the isolates, the efficacy of the phage cocktail on the biofilms increased by 89%. Also, they suggested that using cocktail phages increased activity by expanding the host range and preventing the formation of bacteriophage-resistant mutant bacteria. Notably, the anti-biofilm activity of a cocktail of four phages was not affected by multidrug resistance [43]. In 2017, in a study, researchers isolated bacteriophage AZ1 and tested its anti-biofilm activity against MDR *P. aeruginosa.* The results confirmed the inhibitory and destructive activity of phage AZ1 against *P. aeruginosa* in planktonic and biofilm cells. Researchers suggested that the mechanism of natural phages was to penetrate the biofilm; however, complete eradication may require a combination of phages.[44]. The results of another study on a new phage endolysin, LysPA26, which was tested against planktonic form and *P. aeruginosa* biofilm, showed that the phages had a significant effect on a wide range of MDR Gram-negative bacteria (*Acinetobacter baumannii, Klebsiella pneumoniae, P. aeruginosa*, and *Escherichia coli*) on planktonic form and also eliminated *P. aeruginosa* biofilm. Interestingly, the result showed that LysPA26 had high antibacterial activity against *P. aeruginosa* isolates by influencing the outer membrane under 100 °C heat treatment. The mechanism of biofilm degradation in a concentration-dependent manner by LysPA26 is still unclear and needs further studies [45].

Kwiatek et al. investigated the effects of two bacteriophages MAG1 and MAG4, and their capability to control carbapenem-resistant *P. aeruginosa* in planktonic and biofilm models. It was found that each phage individually affected approximately 50% of *P. aeruginosa* isolates, but when they were used as a cocktail, the anti-biofilm property was increased to 72.9%. Although MAG4 effectively reduced biofilm shortly after the treatment, MAG1 affected biofilm after a more extended period. This study also reported that bacteriophages can utilize three different mechanisms for the eradication of biofilms, including lysis biofilm-forming bacteria by typical phage infection (lysis from within), production of extracellular polymeric substance (EPS) depolymerase, and "lysis from without" that does not need for phage gene expression after absorption. It was also suggested that YefM antitoxin of the bacterial toxin-antitoxin system as a MAG1-encoded homolog might increase the effectiveness of MAG1 over MAG4 [46]. In another experimental study, it was reported that ФKMV, ФPA2, ФPaer4, and ФE2005 phages, either individually or as a cocktail, were capable of destroying biofilm of MDR *P. aeruginosa* isolates in a dose-dependent manner in 24-h assays. In this study, the phage cocktail was not active against two isolates after biofilm formation because of the high production of alginate that its accumulation inhibits phage anti-biofilm activity during 24 h. However, in the conditions with pre-existing biofilm formation, the phages affected these two resistant isolates and eliminated the alginate, which was produced immediately after infection [47]. In another study, the effects of bacteriophages vB-Pa4 and vB-Pa5 on the formation and development of MDR *P. aeruginosa* biofilms were investigated, and the results suggested that bacteriophages almost prevented biofilm formation and also pre-formed biofilms were partially destroyed by phage. Ahiwale et al., in an in vitro study, investigated the management of biofilm produced by antibiotics resistant *P. aeruginosa* using native BVPaP-3 phage. It was found that T7-like lytic phage (BVPaP-3) could inhibit the biofilm formation (three logs) of hospital isolates of *P. aeruginosa.* Also, it was able to disperse pre-made biofilms of all isolates after 24 h [48]. Furthermore, bacteriophage PA1Ø was tested against *P. aeruginosa* biofilm, and it was found that the bacteriophage had lytic properties and required bacterial type IV pili to infect *P. aeruginosa* isolates. Phage PA1Ø had bactericidal activity against a wide range of bacteria (both Gram-positive and Gram-negative), and it was able to eradicate biofilm. This phage can also be introduced as an antimicrobial agent for the treatment of biofilm-associated mixed infections of *Staphylococcus aureus* and *P. aeruginosa*. Due to the probable production of lytic phage enzymes, the mechanism of phage antibacterial action against Gram-positive bacteria may be different from that of *P. aeruginosa*. For example, endolysin can degrade the cell wall of Gram-positive bacteria by destroying peptidoglycan [49].

Based on the above studies, it can be concluded that the identification of new phages can be an excellent alternative to antimicrobial agents for the treatment of MDR *P. aeruginosa* biofilm and may even eradicate the infections caused by MDR *P. aeruginosa* mixed with bacteria in vitro. The results of in vitro investigations can help to increase the application of phages against MDR *P. aeruginosa* nosocomial infections. The use of bacteriophage cocktails can increase anti-biofilm performance and also prevent resistance to bacteriophages. Besides, the production of large amounts of alginate or mature biofilms can inhibit the function of phages, so they should be investigated further in future studies. It should be noted that, due to the very high importance of MDR strains, in this section, we have discussed bacteria with high antibiotic resistance; also, in Table 1, a complete list of studies that have applied bacteriophages to inhibit other *P. aeruginosa* species biofilm is presented.

**Table 1:** Studies using phage therapy to inhibit the biofilm of different strains of *P. aeruginosa*

First author and year	Species	Type of phage	Experimental results	References
Liyuan Mi (2019)	*P. aeruginosa* 1193	Lytic IME180 phage depolymerase	This phage enzyme degraded *P. aeruginosa* exopolysaccharide, enhanced bactericidal activity mediated by serum complement proteins in vitro, and disrupt the bacterial biofilm	[50]
Yangyijun Guo (2019)	*P. aeruginosa* PAO1	vB_PaeM_SCUT-S1 and vB_PaeM_SCUT-S2	These two phages inhibited the growth of bacterium at low multiplicity of infection levels, had good performance both on preventing biofilm formation and eradicating preformed biofilms	[51]
Tomasz Olszak (2017)	*P. aeruginosa* PAO1	O-specific polysaccharide lyase from the phage LKA1	This enzyme reduced *P. aeruginosa* virulence, sensitized this bacterium to serum complement activity, and caused biofilm degradation	[52]
Diana R. Alves (2016)	*P. aeruginosa* PAO1	A cocktail of six specific phage	After 4 h of biofilm contact with the phage suspension (MOI 10), more than 95% of biofilm biomass was eliminated, and 48 h after adding the phage cocktail in the flow biofilm model, the biofilm was dispersed	[53]
Muafia Shafique (2017)	A hospital isolate of *P. aeruginosa*	JHP	This phage reduced biofilm biomass from 2 to 4.5 logs (60–90%) and reduced bacterial load that highlights its potential to prevent biofilm formation from indwelling medical devices	[54]
Ruoting Pei (2014)	*P. aeruginosa* PAO1	Engineered T7 bacteriophage that encode lactonase enzyme	This phage lyses bacteria and expressed quorum-quenching enzymes that inhibited biofilm formation	[40]
A. Phee (2013)	*P. aeruginosa* PA14	JBD4 and JBD44a	These phages significantly reduced the mean percentage of biofilm biomass in 24 and 96-h grown on microplates, but in 24 and 96-h *P. aeruginosa* PA14 biofilms in a root canal model, phage therapy did not affect biofilm inhibition	[55]
Katarzyna Danis-Wlodarczyk (2015)	*P. aeruginosa* PAO1	Bacteriophages KTN6 and KT28	Both of these bacteriophages reduced colony-forming units (70–90%) in 24 h to 72 h *P. aeruginosa* PAO1 biofilm cultures, reduced the secretion of pyocyanin, and pyoverdin, and increased diffusion rate through the biofilm matrix	[56]
Susan M. Lehman (2014)	Clinical *P. aeruginosa* and *Proteus mirabilis*	Novel phages	Phage pretreatment reduced *P. aeruginosa* and Proteus mirabilis biofilm counts by 4 log10 CFU/cm2 and 2 log10 CFU/cm2, respectively, so it is reported that pretreatment of a hydrogel urinary catheter with a phage cocktail can significantly reduce mixed-species biofilm formation by clinically relevant bacteria	[57]
Diana Pires (2011)	*P. aeruginosa* PAO1 and ATCC 10,145	PhiIBB-PAA2 and phiIBB-PAP21),	Both phages after 2 h of infection reduced approximately 1–2 log the biofilm population, and the reduction was further enhanced after 6 h of biofilm infection. *P. aeruginosa* PAO1 showed resistance to phiIBB-PAP21, while phage phiIB-PAA2 for *P. aeruginosa* ATCC10145 continued to destroy biofilm cells, even after 24 h of infection	[58]
P. Knezevic (2011)	*P. aeruginosa* ATCC 9027	δ, J-1, σ-1 and 001A	Phages δ and 001A inhibited bacterial growth and biofilm formation for more than a half at all MOIs, but σ-1 significantly inhibited bacterial growth only at very high MOIs and had no effect on biofilm formation	[59]
Matthew K. Kay (2011)	*P. aeruginosa* PAO1	*Escherichia coli* bacteriophage _W60 and *P. aeruginosa* bacteriophage PB-1	In mixed-species biofilm communities, both of bacterium maintained stable cell populations in the presence of one or both phages	[60]
Weiling Fu (2009)	*P. aeruginosa* M4	*P. aeruginosa* phage M4 and five-phage cocktail from a larger library of *P. aeruginosa* phages	The pretreatment of catheters with phage reduced viable biofilm count by 2.84 log10, and the pretreatment of catheters with the cocktail of phage reduced the 48-h mean biofilm cell density by 99.9%	[61]

## Phage therapy for inhibition of MDR *P. aeruginosa* biofilm: in vivo studies

Various studies have shown advances in using phages against MDR *P. aeruginosa,* which cause chronic otitis media, cystic fibrosis, and burn wounds; however, these studies are limited to pre-clinical evaluations [62–64]. It should be noted that applying phages as antimicrobial agents for the control of pathogens is not a new approach, and it has been used since the phage was discovered. For example, it was used in Eastern Europe and the former Soviet Union for a long time, and even today, phages are used in Georgia to treat some infections [65]. Various studies have been conducted on the use of phages in animal models to evaluate the safety and efficacy of phages to counteract both major Gram-positive and Gram-negative clinical pathogens. Among these, *P. aeruginosa* was particularly important due to its high antibiotic resistance, high mortality, and high production of extensive biofilms in nosocomial infections. Jeon et al. evaluated two novel bacteriophages BФ-R656 and BФ-R1836 in the survival of acute pneumonia mouse models infected with extensively drug-resistant (XDR) *P. aeruginosa* in vitro, in silico, and in vivo. Both phages exhibited potent inhibitory activity and lysed XDR *P. aeruginosa* strains isolated from pneumonia patients. Furthermore, researchers developed two models of in vivo infection. The results demonstrated that BФ-R656 and BФ-R1836 eliminated XDR *P. aeruginosa* strains in *Galleria mellonella* larvae and acute pneumonia mice models. These phages were able to remove the host XDR *P. aeruginosa* biofilms extensively. So, it was suggested that these phages could be used as a biocontrol agent not only to inhibit biofilm formation on medical devices and hospital environments but also to eliminate biofilm-associated infections in the body [65].

In another study, the phage PELP20 was evaluated to counter chronic lung infections with *P. aeruginosa* in a mice model. The results of this study confirmed that the phage had antimicrobial activity on *P. aeruginosa,* and the 3-log phage reduced the biofilm. This indicates that phage PELP20 can kill the biofilm-related bacteria present in the lungs of CF patients [62]. Besides, phage ФPan70, a temperate phage, was used to control MDR *P. aeruginosa* infection in planktonic, biofilm, and mouse burn models. The significant results showed that the phage affected both planktonic and biofilm cells and significantly reduced the bacterial population. Interestingly, the phage resulted in the survival of the burned mice from 80 to 100%. Of note, the reason for the difference in the effect of phage on different isolates was that the phages were strain-specific, and due to the differences in the amount of exopolysaccharide, the effect of phage was different on a variety of biofilms. The researchers speculated that phages could inhibit the spread of bacteria into the bloodstream, and the phages inoculated in the site of infection would confine the high concentration of bacteria and enhance the immune response and cutaneous mastocytosis [66].

Alemayehu et al. tested the effects of two bacteriophages (ФMR299-2 and ФNH-4) to counteract *P. aeruginosa* biofilm in murine lungs; they demonstrated that these two phages killed all clinical isolates (mucoid and non-mucoid isolates). The phage cocktail was effective in killing mucoid and non-mucoid strains growing in the cystic fibrosis bronchial epithelial CFBE41o-cell line. Also, the phage cocktail showed a lethal effect on *P. aeruginosa* in murine lungs, and this bacterium was effectively cleared from the lungs after six hours. They explained that the phages had to penetrate the biofilm exopolysaccharide to be effective; this required a longer time (22 to 24 h) for the phage to be exposed to the biofilm and clear *P. aeruginosa* from infected rat lungs. Also, it has been suggested that the use of diverse phages usually results in the occupation of different bacterial receptors; thus, it requires independent mutations to generate phage resistance. As a result, using different phages in one combination can control the bacterial population if there is no resistance [67]. In 2018, the effect of the cocktail containing six different phages (PYO2, DEV, E215, E217, PAK_P1, and PAK_P4) on the reduction of *P. aeruginosa* biofilm formed in acute respiratory infection model in mice and bacteremia in wax moth (Galleria mellonella) larvae was studied. The results showed that these phages alone could lyse *P. aeruginosa* in both planktonic and biofilm forms. The phage cocktail was also found to be effective on MDR and mucoid phenotype of *P. aeruginosa* isolates. This cocktail was superior to the individual phages in destroying biofilms, and it decreased treatment time in mice. Researchers noted that the phage cocktail reduced different degrees of biofilms; it was able to enter the biofilm, destroy its biomass, and reach the bacteria embedded inside [68]. Another study was performed to evaluate the effect of bacteriophages on MDR *P. aeruginosa* biofilm in a mouse wound model. The results exhibited that the phage cocktail had an inhibitory effect on the biofilm created in mouse wounds, and the count of bacteria was decreased after treatment [69].

Overall, bacteriophages alone and in combination together effectively control XDR and MDR *P. aeruginosa* infections in planktonic and biofilms forms in the animal models. Therefore, human infections that are associated with the *P. aeruginosa* biofilm, particularly XDR and MDR infections, such as lung and wound infections, are associated with therapeutic problems, and they can be the potential future targets of phage therapy. As the results of the in vitro studies show, the use of bacteriophage cocktails in animal models have shown better performance, so it is recommended that it be used more in future studies. Table 2 summarizes some of the studies that used bacteriophages to destroy biofilms of the most important bacterial pathogens. Infection caused by the biofilm of these bacteria, along with the *P. aeruginosa* biofilm, is one of the most important causes of chronic and MDR infections.

**Table 2:** Some studies using phage therapy to inhibit the biofilm of the most important bacterial pathogens

Biofilm forming bacteria	Bacterial Properties	Phage	Outcome	References
*Acinetobacter baumannii*	XDR *A. baumannii*	Phage AB1801	This phage inhibited biofilm formation and reduced preformed biofilms in a dose-dependent manner	[70]
	MDR *A. baumannii*	Phage lysin PlyF307	Treatment with PlyF307 was able to significantly reduce planktonic and biofilm of *A. baumannii,* both in vitro and in vivo	[71]
	*A. baumannii* strain AIIMS 7	Lytic bacteriophageAB7-IBB1	The phage affected *A. baumannii *biofilm formation on an abiotic (polystyrene) and biotic (human embryonic kidney 293 cell line) surface	[72]
	Clinical isolate of *A. baumannii* strain AIIMS 7	Phage AB7-IBB2	The phage could inhibit *A. baumannii *biofilm formation and disrupt preformed biofilm as well	[73]
*Klebsiella pneumoniae*	P DR *K. pneumonia* UA168	The phage KP168	After 48 h of co-cultivation of this phage and the host bacteria at each MOI, the inhibition rates of biofilm were similar, with an average of about 45%	[74]
	MDR *K. pneumonia*	Depolymerase Encoded by Bacteriophage SH-KP152226	This enzyme showed specific enzymatic activities in the depolymerization of the *K. pneumoniae* capsule and was able to significantly inhibit biofilm formation and/or degrade formed biofilms	[75]
	An environmental isolate of *K*. *pneumoniae* ShA2 strain	TSK1 bacteriophage	Post-treatment with TSK1 against different age *K. pneumoniae* biofilms reduced 85–100% biofilm biomass. Pre-treatment of TSK1 bacteriophage against the biofilm of *K. pneumoniae* reduced > 99% biomass in the initial 24 h of incubation	[76]
	MDR *K. pneumoniae* KP/01	Bacteriophage ZCKP1	This phage reduced bacterial counts and biofilm biomass (> 50%) when applied at a high multiplicity of infection (50 PFU/CFU)	[77]
	A clinical strain of *K. pneumoniae*	Bacteriophage Z	Phage Z reduced biofilm biomass twofold and threefold after 24 and 48 h, respectively	[78]
*Staphylococcus aureus*	MRSA	UPMK_1 and UPMK_2 phages	Both bacteriophages were able to destroy biofilms using their lytic enzymes	[79]
	MRSA and MSSA	Bacteriophage CSA13	This bacteriophage removed over 78% and 93% of MSSA and MRSA biofilms in an experimental setting, respectively	[80]
	MRSA ATCC 43,300	Bacteriophage Sb-1	This phage showed a synergistic effect with antibiotics on eradicating MRSA biofilm, direct killing activity on ≈ 5 × 105 CFU/mL persisters cells, and degraded MRSA polysaccharide matrix	[81]
*Escherichia coli*	*E. coli* MG1655 and MDR UPEC strain 390G7	Bacteriophage vB_EcoP-EG1	vB_EcoP-EG1eliminated biofilm of these bacteria. The median biofilm biomass reduction was about 60% and 50% for *E. coli* MG1655 and for clinical isolate 390G7 after 24 h, respectively	[82]
	*E. coli* TG1	T3 bacteriophage	T3 at lower bacteriophage titers (10^3^ PFU/ml) inhibited the production of biofilm	[83]
	*E. coli* 30	vB_EcoM-UFV017 (EcoM017)	This phage reduced the bacterial growth and the quantity of biofilm formed by *E. coli* in 90.0% and 87.5%, respectively	[84]
*Enterococcus faecalis*	*E. faecalis* clinical strains	vB_EfaH_EF1TV	This phage infected *E. faecalis* and degraded biofilm formed by this bacterium	[85]
	VRE *E. faecalis*	Vancomycin-phage EFLK1	This phage, in combination with vancomycin, was synergistically effective against VRE planktonic and biofilm cultures	[86]
	*E. faecalis* and *Enterococcus* clinical isolates	vB_EfaS-Zip and vB_EfaP-Max	The cocktail of these phages reduced 2 and 1 log CFU/mL *E. faecalis* load in biofilms formed in the wound after 3 and 6 h of treatment, respectively, and significantly reduced cell concentration in dual-species biofilm	[87]

MDR, Multi-drug resistant; PDR, Pan-drug resistant; MRSA, Methicillin-resistant *S. aureus*; MSSA, Methicillin-susceptible *S. aureus*; VRE, Vancomycin-resistant Enterococcus, UPEC: Uropathogenic *E. coli*

## Use of combination therapy of antibiotics and bacteriophages to inhibit *P. aeruginosa* biofilm

Applying a combination of different substances with antibiotics to increase their effectiveness on MDR bacteria has received much attention [88]. Furthermore, combination therapy can also be useful in destroying biofilms [89, 90]. Therefore, using various compounds, which are capable of destroying the EPS and increasing the permeability of the biofilm, can boost the antibiotic function, and previous studies have reported that bacteriophages can also enhance antibiotic performance on biofilms. Tamta and his colleagues reported that the use of bacteriophages and antibiotics helped inhibit MDR *P. aeruginosa* biofilm in a patient with diabetes mellitus type 2 who was also diagnosed with relapsing right knee periprosthetic joint infection and chronic osteomyelitis. In this patient, bacteriophage was administered locally during surgery, and a bacteriophage solution was applied every 8 h for five days. The results of isothermal microcalorimetry showed that bacteriophages can help destroy biofilm, and pretreatment of *P. aeruginosa* biofilm with phages, eight hours before colistin exposure, demonstrated the most substantial reduction of biofilm biomass. In this presented case, the combined use of phage, surgery, and conventional antibiotics eradicated the infection, and no phage side effects were observed. However, surgery and antibiotic treatment alone was not sufficient and led to numerous therapeutic failures [91].

**Fig. 1:**
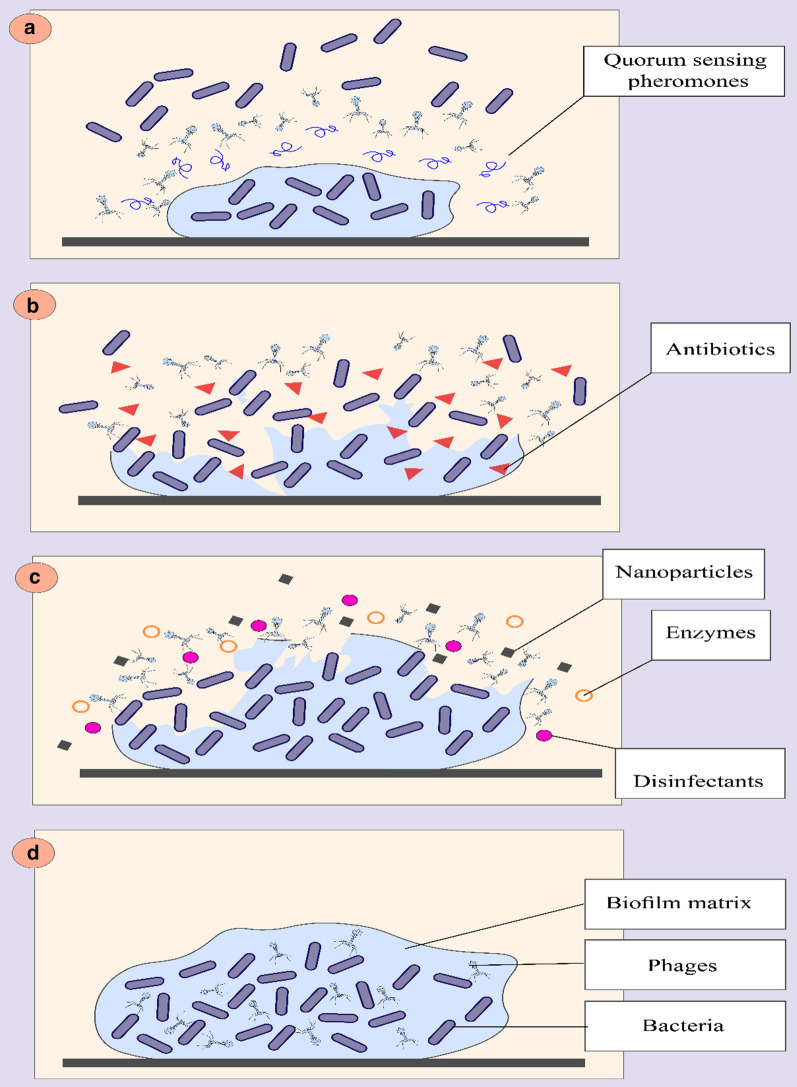
Anti-biofilm mechanisms of bacteriophages. **a** Bacteriophages inhibit biofilm formation by inhibiting quorum sensing and reducing cellular communication. **b** Combined treatments with sequential application of phage and antibiotics have a killing efficacy on *P. aeruginosa* biofilm. **c** Combined use of bacteriophages with molecules with anti-biofilm properties can help biofilm destruction. **d **Bacteriophages can penetrate the inner layers of the biofilm through the biofilm void spaces without destroying the external matrix and replicate in the deeper-layer of biofilm

In another study, a combination of bacteriophage vB_PaM_EPA1 and antibiotics that were selected based on their mechanism of action was used to destroy the biofilm of *P. aeruginosa* [92]. Using bacteriophages and antibiotics alone showed a modest effect on biofilm destruction, but a profound improvement in the killing was observed when applied simultaneously or sequentially. Notably, increasing the concentration of antibiotics when used in combination with bacteriophages did not have a more significant inhibitory effect on the biofilm. This can be related to phage replication inhibition phenomena that protein and DNA synthesis inhibition antibiotics, which results in the suppression of bacteriophages [92–94]. Furthermore, the highest inhibitory effect on biofilm was observed when antibiotics were added sequentially after phage treatment. So, combined treatments with sequential application of phage and antibiotics (Fig. 1) have a better killing effect than the concurrent application. No depolymerase was detected in the bacteriophages used in this study. Therefore, it seems that vB_PaM_EPA1 reaches the bottom layers of the biofilm through the biofilm void spaces [95], thereby replicates in the deeper-layer of biofilm, interrupt the structure of the biofilm and enhance the performance of the antibiotics [92]. In another study, phage PEV20 and ciprofloxacin were used to inhibit the biofilm of *P. aeruginosa* isolated from wound and cystic fibrosis patients. The results showed that combined antibiotic phage treatment enhanced biofilm eradication compared with single ciprofloxacin treatment. This phenomenon can be as a result of the low penetration of this antibiotic into the biofilm and bacteria on the inner layers of biofilm, which show high antibiotic resistance due to low metabolic activity. However, when bacteriophages reduce the integrity of the extracellular matrix, the bacteria in the inner layers of the biofilm are exposed to food and oxygen and become metabolically active; and this can lead to induction of antimicrobial effects of ciprofloxacin and phage [96, 97]. On the other hand, some planktonic bacterial cells were highly susceptible to PEV20. However, after biofilm formation, phage treatment alone was ineffective, and this may indicate that phage monotherapy may lead to resistance to bacteriophages and result in a subsequent increase in bacterial and biofilm density over time; therefore, using cocktails of phages may be helpful in this regard [61]. It should be noted that using a combination of bacteriophages and tobramycin had no change in the inhibitory effect on the bacterial biofilm compared with using tobramycin alone; this may indicate the high penetration of fluoroquinolones into *P. aeruginosa* biofilm [97].

In a similar study, a combination of bacteriophages ((ATCC 12,175-B1 (Pa1), ATCC 14,203-B1 (Pa2), and ATCC 14,205-B1 (Pa11)) and ciprofloxacin was used to destroy the biofilm of different *P. aeruginosa* isolates. The results showed that bacteriophages had a better inhibitory effect on biofilm than antibiotics, and when used in combination, they performed better than applying antibiotics alone. Using phages before antibiotics have the best inhibitory effect on the biofilm because it seems that, in the large bacterial population, phages can increase to a greater extent than the condition of adding phages after antibiotic therapy. Therefore, it seems that the phage application should precede antibiotic treatment. Of note, it is reported that bacteriophages perform better when used in the early stages of biofilm formation, and the large colonies seem to provide spatial refuges that protect the bacterial host from phage infection, which reduces the effect of phage therapy for the treatment of mature biofilms [98]. Chaudhry et al. reported that the combined use of bacteriophages and antibiotics that are commonly used to treat pseudomonas infection leads to pharmacodynamics synergy and could be useful in destroying the *P. aeruginosa* PA14 biofilm [93]. Antibiotics had only a limited effect on the biofilm when used alone. Still, the combination of bacteriophage plus ciprofloxacin and ceftazidime reduced bacterial density below that of the best single antibiotic treatment. On the other hand, the combination of bacteriophages with gentamicin and colistin did not improve the inhibitory effect of antibiotics on biofilms. It should be noted that to simulate the condition of the patient's body and its impact on treatment, the *P. aeruginosa* PA14 was added to the confluent monolayers of human nasopharyngeal cells, and biofilms were formed. All five antibiotics alone inhibited the growth of bacteria on these cells, and simultaneous treatment with the phage and tobramycin significantly increased the efficacy of antibiotics in killing the bacteria. Noteworthy, the results of this study, like previous studies, showed that when bacteriophages were administrated before antibiotics for treating biofilm infections, they could have a better effect than simultaneous administration [93, 99, 100].

Finally, Coulter et al. used a combination of bacteriophage PB-1 and tobramycin to inhibit *P. aeruginosa* PAO1 biofilm. The outcomes of this study showed that combination therapy was not more helpful than the administration of antibiotics alone, and this may be due to the EPS activity that blocks the ability of the phage to access their specific receptors. However, the combined use of PB-1 and tobramycin resulted in a 60% and 99% decrease in tobramycin and phage resistant cell, respectively, compared to the use of tobramycin or phage alone. Thus, combined tobramycin-bacteriophage can significantly reduce the emergence of antibiotic and phage resistant cells; however, the reduction in biomass was dependent on the phage-host system [101]. Therefore, the results of different studies show that the combined use of *P. aeruginosa* specific bacteriophages and effective antibiotics on this bacterium can improve antibiotic efficacy and reduce antibiotic-resistant bacteria in *P. aeruginosa* biofilm. Although most studies have focused on pure bacterial cultures, the presence of polymicrobial biofilm infections, such as wound infection, is very problematic because these biofilms usually have higher antibiotic resistance than monoculture infections [60, 101, 102]. In this regard, the combined use of bacteriophage and antibiotics in future studies for the inhibition of mixed cultures should be considered further.

## Other combination therapies to increase the efficacy of phage therapy on *P. aeruginosa* biofilms

In addition to the combined use of antibiotics and phages, recent studies have used other methods to increase the efficacy of bacteriophages on *P. aeruginosa* biofilms. For example, James and his colleagues engineered an injectable hydrogel capable of encapsulating *P. aeruginosa* (PsAer-9) bacteriophage to treat bone infections caused by this bacterium. Bacteriophages (ΦPaer4, ΦPaer14, ΦPaer22, ΦW2005A) retained their antimicrobial ability after being encapsulated in a hydrogel, and the hydrogel formula controlled the rate of release. Bacteriophage-encapsulating hydrogels effectively lyse *P. aeruginosa* in both planktonic and biofilm phenotypes, in vitro, without affecting the metabolic activity of human mesenchymal stromal cells. Furthermore, hydrogels containing a combination of *P. aeruginosa* bacteriophage significantly (4.7 fold) reduced bacteria counts in the murine radius segmental bone defect in comparison to bacteriophage-free hydrogels, and immune responses to bacteriophages were not observed. This indicates the low toxicity effect of this compound on eukaryotic cells. Notably, in this study, the inhibitory role of bacteriophage-encapsulating hydrogels as prophylaxis for inhibiting the formation of *P. aeruginosa* biofilm in bone was demonstrated. In future studies, the role of this hydrogel in controlling established infections should be considered [103]. Another study reported that the combination uses of vB_PaeP_PAO1-D bacteriophages and chestnut honey could be effective in inhibiting *P. aeruginosa* PAO1 biofilm and can be a promising alternative for topical treatment of wound infection. The results showed that honey and bacteriophages, synergically, increase the antimicrobial effect of each other because they can destroy biofilms using different mechanisms. In this regard, phages infect and destroy bacteria through host-receptor recognition. On the other hand, honey, as another antimicrobial agent, induce destruction in bacteria by other distinct mechanisms such as oxidative stress, osmotic pressure, acidity, hydrogen peroxidase release, and presence of methylglyoxal (MGO). So, honey seems to increase the binding of bacteriophages to their specific receptors by destroying the EPS of the biofilm, and the topical use of phage-honey formulation can be useful for the treatment of chronic wounds since bacteriophages destroy MDR bacteria and honey accelerates the wound healing process [104].

Interestingly, in another study, the combination of bacteriophage and chlorine disinfectants was used to destroy the biofilm of *P. aeruginosa*. Chlorine compounds are one of the most commonly used disinfectants in hospitals, and their combination with bacteriophage was more efficient in reducing *P. aeruginosa* biofilm formation. There were significant differences between combination treatments and single treatment. Chlorination treatments were not capable of destroying the pre-existing *P. aeruginosa* biofilms because the EPS produced in the biofilm prevents the penetration of chlorine into the biofilm. Nevertheless, in combination with bacteriophages, their efficacy increased and eventually led to cell membrane disruption and cell lysis. Therefore, the combined use of bacteriophage and chlorine disinfectants enhances biofilm cell lysis and destruction of *P. aeruginosa* biofilm, which can be used as a promising method for the destruction of bacterial biofilm [105]. Phage, belonging to the Podoviridae family, inspired gold nanoparticles (AuNPs) were another compound reported to have the capability to destroy the *P. aeruginosa* MTCC 728 biofilm. In this study, phage lysate was used to synthesize nanoparticles, and it exhibited higher antimicrobial activity against *P. aeruginosa* compared to bacterial cell-mediated AuNPs. Furthermore, phage inspired AuNPs inhibited the bacterial biofilm formation on the glass surface. AuNPs can inhibit bacterial biofilm due to their toxic effects on bacterial cells, and bacteriophages can help them perform their inhibitory function on the biofilm at a lower concentration. Thus, it is reported that phage inspired AuNPs synthesis may serve as potential therapeutic agents against the biofilm-forming human bacterial pathogens [106].

In another study, QQ lactonase (SsoPox-W2631) were used to destroy the biofilm of clinical isolates and *P. aeruginosa* and increase the effectiveness of bacteriophages and antibiotics on them. The results showed that SsoPox-W263 reduces pyocyanin, protease, and elastase production in antibiotic and bacteriophage-resistant *P. aeruginosa* strains. Furthermore, this enzyme can destroy more than 70% of the biofilms formed in this bacterium and increase the effectiveness of bacteriophages and antibiotics on *P. aeruginosa* biofilms [107]. Finally, another study reported that xylitol could also enhance bacteriophage function for the destruction of stable mixed-species biofilm of *K. pneumonia* and *P. aeruginosa*. Using non-depolymerase producing phage Pa29 was not capable of destroying *P. aeruginosa* biofilm due to limited penetration in the deeper layer. Nonetheless, when Pa29 was used with *K. pneumonia* specific depolymerase producing phage KPO1K2, an enhanced effect was observed on the destruction of the mixed-species biofilm. Therefore, it was suggested that the capsular depolymerase present in *Klebsiella*-specific bacteriophage could increase the permeability of Pa29 due to the destruction of the top layer of the biofilm. Furthermore, xylitol helped Pa29 to destroy the biofilm of *P. aeruginosa*. Still, they were ineffective in mixed-species biofilm destruction because of the distinct spatial distribution pattern adopted by the two organisms in mixed-species biofilm. Hence, the combined use of bacteriophages and xylitol can effectively inhibit *P. aeruginosa* biofilm, and they can be used as one of the therapeutic strategies for tackling chronic infections caused by mixed-species bacterial biofilms [108]. Therefore, in addition to the combined treatment of bacteriophages and antibiotics, which are effective in destroying the *P. aeruginosa* biofilm, the simultaneous use of bacteriophages with natural substances, nanoparticles, and disinfectants can also increase the chances of biofilm destruction of the bacterium (Fig. 1); however, further studies are needed to confirm this finding. Besides, a combination of various natural substances, nanoparticles, and other chemicals, which have been reported in recent studies, may have anti-biofilm properties along with bacteriophages can be used in future studies to destroy the biofilm of MDR *P. aeruginosa*.

## Phage therapy limitation for inhibition of bacterial biofilm

As mentioned in the previous sections, phages are considered as a potential agent for prevention and controlling the biofilm. Still, there are some obstacles in the application of phages for biofilm control.

## Biofilm extracellular matrix limit the diffusion

The extracellular polymeric substance known as EPS consists of 90 percent of the biofilm mass and creates a three-dimension shape of biofilm. EPS prevents the diffusion of the antimicrobial agents through bacteria by covering bacteria cells [109–111]. Furthermore, phages may have initial reversible interaction with some components like capsule polysaccharide, teichoic acids, and lipopolysaccharides, but cell wall components are necessary for irreversible attachment. The existence of the mentioned components in the matrix can limit the phage entrance into biofilm cells [109, 112]. Hu et al., in a study on diffused properties of phages, reported that similar to the antibiotics, slow penetration through biofilm could be a problem for phages. Also, they suggested that phage penetration is dependent on both phage morphology and biofilm density; as the density increase, the diffusion becomes more difficult [113].

## Narrow host range

The molecules on the surface of bacteria, such as lipopolysaccharide and peptidoglycan components, outer membrane proteins, and teichoic acids, could be the attachment sites for the phage tail [114, 115]. Also, the host range is determined by the specificity of phage receptors. Furthermore, some phages have a broad spectrum, while some other phages have a narrow spectrum of host range. Narrow host range could be problematic, especially in polymicrobial biofilms, which are formed on medical devices [115, 116]. Besides, in most of the infectious biofilms, there are two or multiple bacterial species that make the clearance with the phage therapy challenging [117]. Choosing one single phage, which can destroy various extracellular polymers and using it in phage cocktails, can be the right solution against polymicrobial biofilms [115, 118].

## Sub-populate phage resistance in biofilm

Becoming resistant to phages is necessary for the survival of bacteria, and this happens by four different mechanisms. These mechanisms give bacteria adoption to phages and create a phage resistance mutant [109, 119]. There are reports about the rapid growth of phage resistance sub-populate after the primary reduction of biofilm cells that were treated with phages [58, 61, 115, 120]. As Fu et al. studied the formation of biofilm on the hydrogel-coated catheter, they observed that after 24 h in the phage treated group, viable biofilm counts were decreased compared to the untreated group. On the other hand, phage resistant isolates were recovered from the biofilm. Also, when they used a cocktail consisting of five different phages, biofilm cell density was reduced by 99.9%, but they found few isolates resistance to these phages [61]. In another study in two isolates of phage resistant *P. aeruginosa*, genetic analyzes revealed that mutations in *pilT* and *galU* genes are associated with resistance. Furthermore, these genes are associated with pilus motility and LPS formation, respectively. Also, they can act as phage receptors [121].

Additionally, Lacqua et al. found a subpopulation of *E. coli* strain that is resistant to lysis of two phages, and it seems that the biofilm formed by fimbria could be a potential strategy for bacteria to escape phage therapy. They suggested that biofilm formation could be a mechanism for phage resistance alongside with specific mechanisms like changing the receptor or production of DNA restriction enzymes [120]. It seems that the application of phage cocktails could be an excellent solution for preventing this problem [109, 122].

## Reduction in metabolic activity of biofilm bacteria cell

Since phage infection strictly depends on the growth condition of its host, one of the obstacles in the successful use of phage therapy against bacterial biofilm is the reduction of metabolism. Bacteria that are present in biofilms are under nutrient-limited conditions, and they grow slowly [89, 117, 123, 124]. On the other hand, phage infection is dependent on the resources of bacteria, which is directly related to the physiological state, and it is expected that phage infection in planktonic bacteria is more efficient than biofilm bacteria [125]. The result of one study showed that phage infection of *Pseudomonas fluorescens* in planktonic mode increased cell lysis; meanwhile, in biofilm cell lysis, it was notably lower [125]. Also, phage release after the bacterial infection was much higher in the exponential phase rather than in the stationary phase and decline phase [125]. In another study by los et al., it was revealed that starvation of phage could cause severe inhibition of phage lytic development [123].

Furthermore, Cerca et al. studied *Staphylococcus epidermidis,* and they suggested that lysis of bacteria in biofilm is slower than planktonic culture, and it is probably due to low metabolism in biofilm cells. Nevertheless, cells are more sensitive in the exponential growth phase rather than the stationary growth phase in planktonic mode [118]. In both Sillankorva et al. and Cerca et al. studies, biomass reduction, was equal in biofilm and planktonic mode, and it seemed that biomass reduction was dependent on cells' physiological state rather than biofilm phenotype [117, 118, 125].

## Inhibition of phage infection via* Quorum sensing (QS) in biofilm*

Many bacteria use QS as a communication system between cells with extracellular chemical molecules called auto-inducers. QS allows bacteria to coordinate gene expression and density of cells population [126, 127]. Furthermore, it is well known that QS controls biofilm formation, growth, and dispersion, and there are suggestions for inhibiting biofilm formation by disabling QS [128]. In a study on the QS effect on bacteria’s antiphage mechanism, the results demonstrated that in response to N-acyl-L-homoserine lactone (AHL), which is the QS signal, reduction of λ phage receptor happened in *E. coli*. This led to an increase of uninfected bacteria after the phage challenge, and AHL could reduce the chance of infection in a broader range of phages [129]. Notably, the QS effect against phages needs more investigations and descriptions for other bacteria species [109]. Overall, QS could create biofilm and use two different methods for inhibiting the phage effect on biofilm. First, it can reduce metabolic activity and recourse optimization in the biofilm that leads to decreased phage infection efficiency; second, it can regulate antiphage mechanisms [109].

## Conclusion and perspective

Biofilm is one of the leading causes of antibiotic resistance and chronic infections. Because of the inefficacy of antibiotics to inhibit bacterial biofilm, new strategies are needed to combat it. Recent studies have identified phage therapy as one of the effective methods for the destruction of *P. aeruginosa* biofilm. As noted above, there are still limitations to the widespread use of phage therapy, and a focus is needed to address these issues in future studies. The use of new strategies to enhance the efficacy of bacteriophages on the biofilm of *P. aeruginosa* is helpful. Furthermore, it is recommended that future studies use phage therapy to prevent chronic infections caused by *P. aeruginosa* biofilm so that hopefully it paves the way for more using this therapeutic approach.

## Data Availability

All data were included.
